# Experiences Providing Medical Assistance during the Sewol Ferry Disaster Using Traditional Korean Medicine

**DOI:** 10.1155/2017/3203768

**Published:** 2017-11-07

**Authors:** Kyeong Han Kim, Soobin Jang, Ju Ah Lee, Bo-Hyoung Jang, Ho-Yeon Go, Sunju Park, Hee-Guen Jo, Myeong Soo Lee, Seong-Gyu Ko

**Affiliations:** ^1^Department of Preventive Medicine, College of Korean Medicine, Woosuk University, Jeollabuk-do, Republic of Korea; ^2^KM Fundamental Research Division, Korea Institute of Oriental Medicine, Daejeon, Republic of Korea; ^3^Internal Medicine, College of Korean Medicine, Gachon University, Seongnam, Republic of Korea; ^4^Department of Preventive Medicine, College of Korean Medicine, Kyung Hee University, Seoul, Republic of Korea; ^5^Internal Medicine, College of Korean Medicine, Semyung University, Chungbuk, Republic of Korea; ^6^Department of Preventive Medicine, College of Korean Medicine, Daejeon University, Daejeon, Republic of Korea; ^7^Department of Pharmaceutical Affairs, The Association of Korean Medicine, Seoul, Republic of Korea; ^8^Clinical Research Division, Korea Institute of Oriental Medicine, Daejeon, Republic of Korea

## Abstract

**Background:**

This study aimed to investigate medical records using traditional Korean medicine (TKM) in Sewol Ferry disaster in 2014 and further explore the possible role of traditional medicine in disaster situation.

**Methods:**

After Sewol Ferry accident, 3 on-site tents for TKM assistance by the Association of Korean Medicine (AKOM) in Jindo area were installed. The AKOM mobilized volunteer TKM doctors and assistants and dispatched each on-site tent in three shifts within 24 hours. Anyone could use on-site tent without restriction and TKM treatments including herb medicine were administered individually.

**Results:**

The total of 1,860 patients were treated during the periods except for medical assistance on the barge. Most patients were diagnosed in musculoskeletal diseases (66.4%) and respiratory diseases (7.4%) and circulatory diseases (8.4%) followed. The most frequently used herbal medicines were Shuanghe decoction (80 days), Su He Xiang Wan (288 pills), and Wuji powder (73 days).

**Conclusions:**

TKM in medical assistance can be helpful to rescue worker or group life people in open shelter when national disasters occur. Therefore, it is important to construct a rapid respond system using TKM resources based on experience.

## 1. Background

A disaster is a social emergency that occurs when damage to the lives and possessions of people in a nation outweighs the capacity of the society to absorb the damage [1]. Worldwide, humans have experienced numerous disasters throughout history, and we have continuously made efforts to reduce the damage from various disasters, although there are differences among individuals, cultures, and societies [2]. In the past, disasters mostly took the form of natural disasters, although we have seen the possibility of man-made disasters increasing as societies have become more developed, with advances in industry, transportation, and higher mobility. Therefore, the response to man-made disasters has become more important than ever before [3].

Also disasters have become complex and take various forms, and it has become more difficult for today's disasters to be efficiently dealt with using the existing response paradigm [4]. In particular, it can be difficult to use medical devices that are essential to preserve life in an emergency due to a lack of electricity, gas, tap water, medical products, and effective communication [5]. Groups of people who are evacuated from an emergency area into an open shelter with poor hygiene can experience minor symptoms, such as colds, diarrhea, and myalgia [6].

Korea has experienced various man-made and complex disasters during the last decades. These disasters have included subway arson: “a fire broke out in subway and 192 people died in Daegu on February 18, 2003,” the collapse of a department store: “the Sampoong Department Store collapsed and 501 people died in Seoul on June 29, 1995,” a bridge collapse: “the Sungsu bridge was broke down and 32 people died in Seoul on October 21, 1994,” and the sinking of a ship: “Suhae Ferry sank and 292 people died in Busan on October 10, 1993” and have had a significant impact on Korean society. Recently the Sewol Ferry sinking accident occurred in Korea and was a devastating disaster for the nation in 2014.

Particularly in Sewol Ferry disaster, there had been two kinds of medical assistance operated separately: conventional medicine and TKM service. The goal of this study was to survey examples of medical assistance involving TKM provided to the Sewol Ferry sinking victims and to further explore the possible role of TKM in medical assistance.

## 2. Methods

### 2.1. Accident Outline

The Sewol Ferry (a 6,000 ton passenger ship) was carrying 476 passengers. It departed for Je-Ju Island from Incheon at 9:00 PM on April 15, 2014. The ferry began to lose its balance on the sea 1.7 miles away from the southwest coast of Gwangmae-do, Jindo-gun, Jeollanam-do, at 8:50 AM on April 16, 2014, and completely sank on April 18, which led to 295 passenger deaths and 9 missing passengers. The Ministry of Health and Welfare and the Central Emergency Medical Center were first contacted about the accident through the media at 9:30 AM on the 16th before a disaster control room was opened. They reinforced their response efforts by installing the first on-site emergency tent at 9:40 AM and supplying medical products as well as other facilities. In the first rescue, 47 passengers were sent to the on-site emergency tent until 3:00 PM for triage based on the severity of their injuries and were then transported to local medical institutions. With confirmation that there would be no more rescues, efforts to provide medical assistance began [4].

### 2.2. Installation of On-Site Tents for the Administration of Korean Medicine

**Figure 1:**
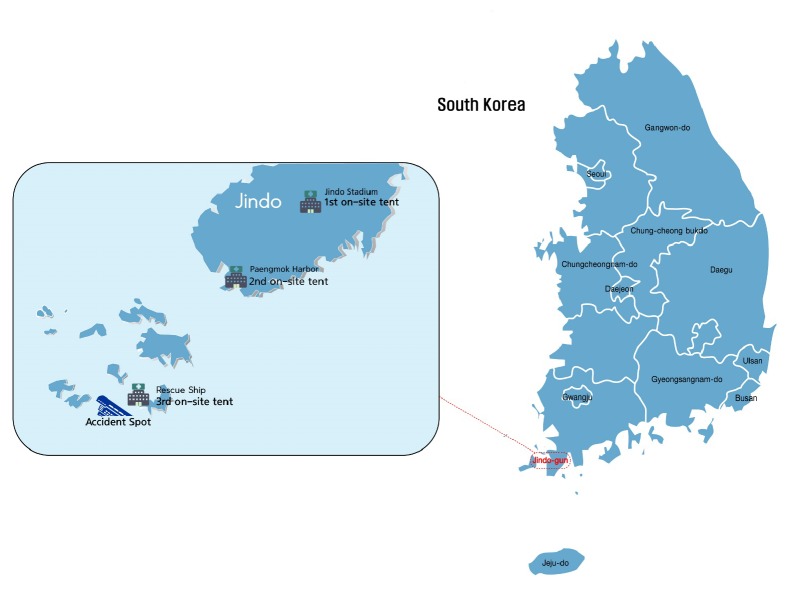
Distribution of on-site tents in Jindo.

**Figure 2:**
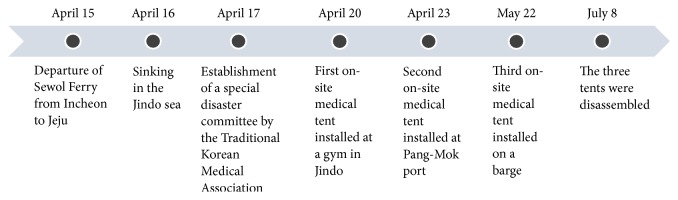
Process for providing medical assistance following the Sewol Ferry disaster using traditional Korean medicine.

There was sporadic mobilization of medical assistance from national Korean medicine doctors and institutions beginning at the time of the accident. On April 17, the day after the accident, the AKOM formed a special disaster committee to integrate Korean medicine, medical volunteers, and institutions. The AKOM consulted with the Ministry of Health and Welfare to install the first on-site tent at Jindo public gym, where the victims' families were, and dispatched Korean medicine doctors and assistants [7]. Beginning on April 20, the on-site tent was operated by the Central Emergency Medical Center, which reported on the medical activities twice a day to the medical assistance board of the Ministry of Health and Welfare. At the request of the Center, the second on-site tent was installed on April 23 at Pang-Mok harbor, where the victims' families and rescue workers were on stand-by. On May 22, the third on-site tent was installed to provide medical assistance to rescue workers on the barge. On July 8, the assistance work being performed at the three on-site tents was transferred to the public medical clinic in the Jindo district, and the tents were removed [8] (Figure 1). The process for providing medical assistance is shown in Figure 2.

### 2.3. Patients

Anyone, victims' families, rescue workers, volunteers, and so on, who felt abnormal in the field, could use the on-site tents without restriction. Also, both conventional medicine and traditional Korean medicine tent were installed, so it was able to choose where patients wanted to go. The data of the patient's age and gender using traditional Korean medicine tent were not provided by the government due to the protection of personal information.

### 2.4. Operation of On-Site Tents

The first tent was installed at a Jindo public gym, where most of the victims' families waited to receive treatment. Medical professionals treated 1,230 people or 15.4 people per day on average. Medical professionals at another tent installed at Pang-Mok harbor treated 630 people or 8.3 people per day on average. Additionally, three other on-site tents were installed at Pang-Mok harbor to supply conventional medical treatment to a total of 3,288 people. The number of patients treated was far lower than the number treated at the two other on-site tents, which were installed for no longer than 23 days, where 34.3 people and 62.9 people were treated per day on average [4], but the number of patients was similar to the number treated at an on-site tent that was installed for no longer than 90 days and provided Korean medicine, treating 11.6 people per day on average [4].

### 2.5. Supply of Medical Services

The AKOM mobilized volunteer Korean medicine doctors and assistants, dispatching seven Korean medicine doctors along with four assistants on average in three shifts within 24 hours. Total 67 Korean medicine doctors participated (male : female = 46 : 21). Six Korean medicine university hospitals (Kyung Hee, Won Gwang, Domg Guk, Daegu Hanny, Dong Shin, and Dong Eui) and four private Korean medicine hospitals (Jaseng, Chungyeon, Dongseo, and Jooenraphas) dispatched medical staffs regularly. Also doctors of private Korean medical clinics participated. All of Korean medicine doctors had national license and more than 5-year experience. Medical supplies, products, and other consumables were purchased voluntarily by members of the AKOM or through fund raising. At the medical office, Korean medicine doctors diagnose and treat patients independently. There was no specific guideline for treatment and doctor decided the treatment method, herbal medicine individually. Acupuncture, moxibustion, cupping, pharmacoacupuncture, and chuna were performed, and patching and taping treatments were administered. Herbal medicines, including decoctions, powders, pills, and extracts, were prescribed depending on symptoms. The list of herbal medicines supplied at that time can be found in Table 3.

The questionnaire was not implemented because of the hectic situation directly after the disaster. Also the patients' residential areas were scattered throughout the country, and treatment effect of each patient could not be judged sufficiently.

### 2.6. Ethics and Consent

We collected the medical records with the cooperation of both the AKOM and Ministry of Health and Welfare. In this study, we could not receive an original individual medical chart. We received, however, the medical activities that were reported twice a day to the medical assistance board of the Ministry of Health and Welfare. An institutional review board at the Kyung Hee University approved this study (number KHSIRB-16-049) for use of the medicinal records.

## 3. Results

### 3.1. Medical Activities

Excluding the medical assistance provided on the barge, for which there were no medical records, a total of 1,860 patients were treated during the disaster period. The first on-site tent installed at the Jindo public gym treated 1,230 patients, and the second on-site tent at Pang-Mok harbor treated 630 patients. Regarding the medical activities at the first on-site tent, 453 patients (36.8%) were treated starting from the day of the accident on the 5th through the 14th; 341 patients (27.7%) were treated from the 15th through the 30th; and 436 patients (35.5%) were treated after the 30th (Table 1).

At the medical office, Korean medicine doctor diagnosis and treat patients independently. There was no specific guideline for treatment and doctor decided to treatment method, herbal medicine individually. Acupuncture, moxibustion, cupping, pharmacoacupuncture, and chuna were performed, and patching and taping treatments were administered. Herbal medicines, including decoctions, powders, pills, and extracts, were prescribed depending on symptoms. The list of herbal medicines supplied at that time can be found in Table 3.

### 3.2. Chief Complaints of Patients Involved in the Sewol Ferry Disaster

There were 1,003 diagnostic medical records from the first on-site tent in the Jindo public gym, and the diagnoses are shown in Table 2. The major reported symptoms included musculoskeletal disorders (64.1%), followed by gastrointestinal disorders (8.7%) and psychological problems (8.7%), respiratory disorders (7.9%), neurological disorders (6.1%), exhaustion (3.4%), and circulatory disorders (0.2%), as well as other symptoms (1.2%). The detailed symptoms were as follows: (1) lower back pain (16.6%), shoulder pain (11.8%), neck stiffness (11.2%), lower limb pain (10.3%), knee pain (4.7%), upper limb pain (3.5%), sprain (3.2%), hip joint pain (1.9%), and other (1.1%) in the category of musculoskeletal disorders; (2) dyspepsia (5.8%), abdominal pain (1.3%), constipation (1.0%), and diarrhea (0.6%) in the category of gastrointestinal disorders; (3) depression (5.3%), anxiety (4.7%), and insomnia (1.9%) in the category of psychological problems; (4) acute pharyngitis (3.3%), common cold (2.6%), and acute rhinitis (2.0%) in the category of respiratory disorders; and (5) headache (4.3%), dizziness (13.0%), and nausea (0.5%) in the category of neurological disorders. In addition, in the category of miscellaneous symptoms, pruritus (0.3%), edema (0.2%), superficial burns (0.2%), tinnitus (0.1%), acute hemiplegia (0.1%), poststroke syndrome (0.1%), lip tremor (0.1%), and hand tremor (0.1%) were recorded. The records on major symptoms of other on-site tents were not written in report.

### 3.3. Herbal Medicines Used for Sewol Ferry Disaster Medical Assistance

It was reported that herbal medicines were prescribed at the first on-site tent at the Jindo public gym from April 20 to May 16. The formulae of the herbal medicines included decoctions, pills, and powders. The most frequently used herbal medicines were Shuanghe decoction (80 days), Su He Xiang Wan (288 pills), and Wuji powder (73 days). The details of the amounts used and the effectiveness of every herbal medicine are presented in Table 3.

## 4. Discussion

The major symptoms that were treated at the conventional medicine on-site tents were the musculoskeletal system (54.8%), followed by the digestive system (6.9%) and the respiratory system (6.1%) [4], whereas the major symptoms treated at the Korean medical on-site tents were the musculoskeletal system (61.1%), followed by the digestive system (8.7%). The only difference was that the Korean medicine on-site tents treated mental disorders (5.1%) and exhaustion (2.5%), whereas the medical on-site tents treated dermatological diseases (11.1%). The dermatological diseases that required rapid treatment were given preference at the medical on-site tents, and Korean medicine was chosen for the treatment of psychological and physical symptoms, such as mental disorders and exhaustion. At 1st on-site tent, patients were mostly family of victims and their main symptoms were cold, diarrhea, and decreased physical and psychological discomfort. At 2nd on-site tent and 3rd on-site tent, mainly ground rescue agents and divers those who suffered from cold and musculoskeletal disorders used TKM service. The reason for the similar patterns of both daily patients and their symptoms between the Korean medical on-site tents and medical on-site tents was that most of the patients were family members or relatives of dead or missing victims, not the victims themselves. These trends indicate that Korean medicine may play a role in addressing medical needs other than surgical needs during disaster situations. This indication was supported by a previous study regarding the effects of Korean medicine treatments following disasters to treat cold symptoms, diarrhea, and myalgia [6]. Given that there must be other medical needs in addition to the need for first-aid treatment during disasters [7, 9, 10], the role of Korean medicine treatments is important.

There was confusion among the government, the AKOM, and volunteers regarding the installation and operation of an on-site tent in the early stages of the Sewol Ferry accident response because Korean medicine is not included in the national disaster medical assistance system. The actual work at the on-site tent began four days after the accident, and all the staff who were mobilized were volunteers. Any medical supplies, products, and consumables used were provided by Korean medicine doctors who donated money for assistance. Some beds and desks were provided by a public health clinic in Jindo district. Korean medicine doctors sent decoctions and pills for prescriptions for expected disorders to the on-site tents, with Shuanghe decoction being the most popular prescription. The AKOM subsequently minimized the possibility of any overlapping prescriptions provided in large quantities through daily checks and online reports of deficient prescription supplies. In addition, no standardized form for medical records was prepared, and every doctor used his or her own form; thus, all of the records were different from one another. The AKOM later provided a medical record form only to record simple medical information, but it was not designed to record information reflecting the disaster situation itself. Moreover, there was no collection of basic information such as the sex and age of the victims and their families who received treatment. Therefore, it was apparent that the use of such information was limited.

No education was implemented before the medical staff were dispatched, and they therefore failed to adequately respond to the typical PTSD symptoms, including extreme physical and mental anxiety and drastic deterioration of physical status. Furthermore, medical products and consumables were not properly supplied due to a lack of consideration of the nature of medical needs following the disaster.

Under these circumstances, the second on-site tent was installed at Pang-Mok to meet the growing need for Korean medicine at the site after installation of the first on-site tent at the gym in the Jindo district. The third on-site tent was installed on a barge because of health concerns raised about the staff, since a diver who was conducting a search on May 6, 2014, died due to loss of consciousness.

To prepare to provide Korean medicine assistance more rapidly and efficiently in a similar situation, we suggest the following policies. Since the Sewol Ferry accident, the national safety authority was launched in November 2014 to meet nationwide need for reform of the national disaster response system. The national safety authority has integrated the existing response systems, which were divided into systems for land, sea, natural, and social disasters, in an attempt to establish itself as a control center for national disasters [11]. However, Korean medicine is currently not included in the national disaster response system, which could again lead to confusion in terms of the installation and operation of on-site tents and other responses to incidents such as the Sewol Ferry accident. Therefore, the inclusion of Korean medicine in the national disaster response system is highly recommended. To this end, the government should develop an integrated medical assistance model including Korean medicine in the disaster response, and based on this model, local governments should work with related organizations in their regions to establish a disaster response plan and conduct joint training. Regarding medical products and supplies, they must be listed by disaster in advance and primarily supplied to the disaster area; support could then be provided from the surrounding areas when supplies are low.

In a disaster situation, medical doctors can be responsible for first-aid treatment of patients, and Korean medicine doctors can treat rescue workers. There is a report showing that prescriptions of Maimendong decoction and Xiao Qing Long Tang were effective for individuals at a rescue site who had a runny nose, stuffy nose, or dry cough, and these prescriptions can be used instead of antihistamines, which have side-effects that reduce attention and promote sleepiness [6]. In a group facility, such as a shelter with poor hygiene, minor symptoms, including chills, fever, cough, runny nose, diarrhea, constipation, and insomnia, are frequently prevalent, and Korean medicine treatments could help to alleviate these symptoms.

There are some limitations in this study, and overcoming these is prerequisites in order that TKM could be included in disaster medical system. First of all, the effectiveness of TKM treatment was not measured because of its special and urgent situation. Although detailed investigation might have been difficult, appropriate and simple evaluating measurements for disaster should be applied. Second, many medical data were missing in report and statistical analyses could not be done. From now on, medical records in disaster situations need to be fully recorded and archived. Third, a scheme for disaster response education with certification must be developed for Korean medicine doctors, and these doctors must be regularly required to participate in refresher training courses.

Although this limitation, the effect of the treatment was indirectly measured in two respects.

First, patients treated in traditional Korean medicine on-site tent in Jindo public gym (15.4 people per day on average) and it is similar to number treated conventional medicine on-site tent (11.6 people per day on average) which operated for a similar period. The use of more patients compared to conventional medicine on-site tent can indirectly show the traditional Korean medicine treatment's therapeutic effect. Also considering that the victim's family mainly lives in Jindo public gym, it can be assumed that traditional Korean medical assistance could contribute to group life in a disaster situation. Acupuncture and moxibustion treatments are timely, handy, and easy to use. In addition, they have the merit of providing medical services without the need of any special medical facilities as far as the operator doctor is skilled in emergency. Thus, they can be applied immediately in a disastrous situations.

Secondly, the first on-site tent in Jindo public gym was installed by the AKOM, but the second on-site tent in Pang-Mok harbor and the third on-site tent in the barge were all installed at the request of the government. This indicates that the patients were satisfied with the first on-site tent at the time and requested to install additional on-site tents [7]. Therefore, it can be assumed that the patient's satisfaction of traditional Korean medical assistance was high. The use of Korean traditional medicine assistance during Sewol disaster was similar to that of conventional medicine assistance, and the satisfaction of the patients was also high. Therefore, it is possible to confirm the possibility of traditional Korean medicine assistance in a disaster situation. Particularly, as the case of Sewol disaster, emergency patients rarely occurred, have to live a group life, and need medical assistance for rescue workers, traditional Korean medicine assistance also needed with conventional medicine assistance.

In Korea, especially, Korean medicine is included in the public medical system among the medical system, so it can be considered that it is easy to provide medical service in the state of emergency. In the future, it will be necessary to systematically manage the traditional Korean medicine assistance within the national disaster medical system. In addition, it will be necessary to nurture a Korean medicine doctor who has expert knowledge to cope with the disaster situation.

## Figures and Tables

**Table 1:** Operational results of medical assistance provided using traditional Korean medicine following the Sewol Ferry disaster.

Classification	Number of patients (%)
Total treated patients (2014. 4. 20-7.8)	1,860 (100.0)
Site distribution	
1st on-site tent (2014. 4. 20-7.8)	1,230 (66.1%)
2nd on-site tent (2014. 4. 23.-7.8)	630 (33.9%)
3rd on-site tent (2014. 5. 22.-7.8)	Not reported
Period distribution^*∗*^	
Acute (5th–14th)	453 (36.8%)
Subacute (15th–30th)	341 (27.7%)
Chronic (after 30th)	436 (35.5%)

^*∗*^1st on-site tent/incident onset.

**Table 2:** Chief complaints of patients treated with traditional Korean medicine at the first on-site tent.

Chief complaints	Number of patients (%)
Musculoskeletal disorders	
Neck stiffness	112 (11.2)
Sprain	32 (3.2)
Low back pain	166 (16.6)
Upper limb pain	35 (3.5)
Knee pain	47 (4.7)
Shoulder pain	118 (11.8)
Lower limb pain	103 (10.3)
Hip joint pain	19 (1.9)
Others	11 (1.1)
Subtotal	643 (64.1)

Gastrointestinal disorders	
Dyspepsia	58 (5.8)
Constipation	10 (1.0)
Abdominal pain	13 (1.3)
Diarrhea	6 (0.6)
Subtotal	87 (8.7)

Respiratory disorders	
Acute pharyngitis	33 (3.3)
Common cold	26 (2.6)
Acute rhinitis	20 (2.0)
Subtotal	79 (7.9)

Psychological problems	
Anxiety	47 (4.7)
Depression	53 (5.3)
Insomnia	19 (1.9)
Subtotal	87 (8.7)

Neurological disorders	
Headache	43 (4.3)
Nausea	5 (0.5)
Dizziness	13 (13.0)
Subtotal	61 (6.1)

Exhaustion	34 (3.4)

Circulatory disorders	2 (0.2)

Others	
Tinnitus	1 (0.1)
Acute hemiplegia	1 (0.1)
Poststroke syndrome	1 (0.1)
Edema	2 (0.2)
Pruritus	3 (0.3)
Lip tremor	1 (0.1)
Hand tremor	1 (0.1)
Superficial burns	2 (0.2)
Subtotal	12 (1.2)

Total	1,003 (100)

**Table 3:** Herbal medicines used in the medical assistance provided following the Sewol Ferry disaster.

Classification		Herbal medicine	Efficacy^*∗*^	Amount
Decoction		Shuanghe decoction	Fragile habitus, fatigue recovery, overwork, during illness or convalescence	80 days
		Guy ZhiMa Huang Ge Ban Tang	Cold, cough, itch	10 days
		Xiang Sha Ping Wei San	Anorexia with dyspepsia, gastric atony	41 days
		Guizhi decoction	Initial cold with physical deterioration	12 days
		Ganmaidazao decoction	Sudden loss of consciousness and convulsions	22 days
		Ganjie decoction	Swelling and pain in throat	16 days
		Insampaedok-san	Fatigue cold, fever, headache	16 days
		Xiao Qing Long Tang	Bronchitis, bronchial asthma, runny nose, cough with dilute phlegm, rhinitis	31 days
		Huoziang-Zhengqi powder	Summer cold, anorexia caused by heat, diarrhea, tiredness	1 day
		Modified Xiaoyao powder	Shoulder pain, fatigue, and anxiety of weak woman	17 days
		Jiawei Wendan decoction	Insomnia with stomach weakness, nervousness	9 days

Herbal medicinal products	Pill	Su He Xiang Wan	Feeling heavy with worries, anger, suddenly falling down	288 pills
		Gongjin-Dan	Feeling of helplessness, physical degradation, dizziness due to deterioration of liver function, headache, chronic fatigue	137 pills
		Tianwangbuxin Dan	Insomnia, anxiety, thirsty, palpitation, shortness of breath, neurasthenia, forgetfulness, hot chest	107 pills
		Uwhangchungsimwon	Palpitation, nervousness, autonomic imbalance	53 pills

Herbal medicinal products	Powder	Buzhongyiqi decoction	Fragile habitus, tiredness, convalescence, anorexia	2 days
		Five Retention powder	Gastroenteritis, low back pain, neuralgia, arthralgia, feeling of cold, common cold	73 days
		BanxiaBaizhuTianma Decoction	Stomach weakness, feeling of cold in lower limbs, dizziness, headache	6 days
		Shensuyin	Cold, cough	2 days
		Taorenchengqi decoction	Neurasthenia, hypertension	16 days
		Xiang Sha Ping Wei San	Anorexia with dyspepsia, gastric atony	14 days
		JiuweiQianghuo decoction	Headache caused by cold, neck stiffness, fever, arthralgia	14 days

^*∗*^Data from the Korea Pharmaceutical Information Center, http://www.health.kr/.
